# The effects of gamification on antimicrobial resistance knowledge and its relationship to dentistry in Saudi Arabia: a randomized controlled trial

**DOI:** 10.1186/s12889-020-08806-2

**Published:** 2020-05-13

**Authors:** Khalid Aboalshamat, Amjad Khayat, Ragheb Halwani, Ammar Bitan, Ryyan Alansari

**Affiliations:** 1 Dental Public Health Division, Preventative Dentistry Department, College of Dentistry, Umm Al-Qura University, Makkah, Saudi Arabia; 2 Medicine and Medical Science Research Center, Deanship of Scientific Research, Umm Al-Qura University, Makkah, Saudi Arabia; 3 College of Dentistry, Umm Al-Qura University, Makkah, Saudi Arabia

**Keywords:** Gamification, Antimicrobial resistance (AMR), Oral health, Knowledge, Randomized controlled trial

## Abstract

**Background:**

Antimicrobial resistance (AMR) has reached alarming levels and is considered to be a worldwide public health problem. One of the most significant factors contributing to the spread of AMR is the lack of proper knowledge about the use of antibiotics, which are being used more frequently in dentistry. Recent studies have found that gamification shows promising results for helping the average person improve their knowledge about health and may also be used to boost knowledge about AMR among the public. This study aimed to assess the effects of gamification on AMR awareness, using a board game to promote knowledge about AMR among the public in Saudi Arabia.

**Methods:**

Using a single-blinded parallel group randomized controlled trial design, 94 volunteers were recruited and randomized into two groups. The study group received information about AMR by playing a board game, while the control group received the same information given in a conventional lecture. The participants were evaluated three times: (T1) before the intervention, (T2) immediately after the intervention, and (T3) one month after the intervention for follow-up to evaluate their retention of the information.

**Results:**

Results showed that there were significant improvements (*p* <  0.05) in knowledge scores for T2 and T3 in comparison to the T1 baseline scores in both groups. However, the knowledge scores also relapsed significantly from T2 to T3 in both groups. Nevertheless, the difference in knowledge score T1 to T3 was significantly higher in the study group in comparison to the control group, and the participants had higher mean scores to use the game as health promotion method.

**Conclusions:**

Gamification using a board game can significantly improve AMR knowledge, with better retention than conventional lecture. It is a promising method for boosting public knowledge about AMR and its relationship to dentistry.

**Trial registration:**

ISRCTN registry: ISRCTN15884410 (retrospectively registered 26-October-2019).

## Background

The term “gamification” has trended to more widespread use in the past few years due to the contribution of gaming in making the educational process more exciting and interesting for students [1]. Gamification describes the use of a game for educational purposes, combined studies with entertainment to increase participant motivation and engagement [2].

Several studies have shown the efficiency of gamification as an approach to improving student understanding in different study fields such as engineering [3] and medicine [4]. In addition, gamification has been helpful in health promotion areas, such as helping with diet modification and exercise promotion [5]. However, the area of gamification has only recently emerged in health care.

Only a few studies have used gamification in the health promotional field related to dentistry, but two studies were found where gamification improved oral hygiene knowledge and attitudes among children in India [6, 7]. The first one was a quasi-experiment that used the Snakes and Ladders board game and found improvement in knowledge immediately after participants had played the game for seven days [6]. The second was a randomized controlled trial (RCT) that used the dot game and found more improvement at a three-month follow-up in the study group as compared to the control group that had only conventional educational methods [7].

Only one study used gamification in the health field in Saudi Arabia [8]. This study assessed the satisfaction of dental students with gamification for improving their academic writing skills. The study results showed that the writing skills of participants were significantly improved after the intervention, despite having low satisfaction levels with the game. Thus, gamification seems to be an emerging and promising avenue for use in health promotion in general and oral health in particular.

One area in the health care field that stands to benefit from better education efforts is with regard to antimicrobial resistance (AMR), which is the modification to bacteria that occurs in response to the overuse of antibiotic (AB) treatment and has led to the ABs becoming ineffective [9]. The World Health Organization (WHO) also stated that AMR has reached alarming levels and is considered to be a worldwide public health problem with the ability to affect anyone [9]. The use of AB treatments without a prescription was estimated to be 58% in Asia, 47% in southern Europe, 30% in eastern Europe, 25% in South America, and 39% in the Middle East [10]. These high numbers of AB misuse might be due to a lack of proper knowledge, as suggested by a cross-sectional study that assessed AB knowledge and attitudes in three countries, including Saudi Arabia [11]. The study results showed a correlation between low levels of knowledge and AB self-medication and showed that 48% of Saudis have taken ABs without a prescription [11]. In fact, a recent systematic review indicated that dental treatment often involves the misuse of ABs as well [12]. Thus, AMR awareness interventions are essential to boost knowledge and improve attitudes. A recent systematic review evaluating the effectiveness of interventions targeted at proper AB use and AMR knowledge concluded that the previous interventions were of poor quality and targeted only high-income countries, which prompted a drive to conduct well-designed interventions [13].

Gamification seems to be an innovative method for helping to boost AMR awareness. To the best of our knowledge, there has been only one recent study that used gamification as a method of improving AMR awareness [14]. This study was conducted on 153 children in the United Kingdom, using three different web-based games (e-Bug) aimed at improving their AMR knowledge. The results showed that while all three games improved knowledge, the level of improvement and level of enjoyment varied according to the game type. However, the assessment was conducted immediately after playing a game and without a control group for comparison. Also, this study involved only children, while problems with AB misuse more likely arise with adults, who have more access to ABs and are responsible for giving ABs to the children. Also, developing such web-based games can be costly, particularly in comparison to other game methods, such as board games.

As literature above showed public low AMR knowledge, Thus, this study aimed to assess the effects of gamification with board games (as innovative method) to improve AMR knowledge in relation to dentistry in Saudi Arabia.

## Methods

### Study design and participants

This study was conducted using a single-blinded parallel group RCT design, where the participants in the study group (SG) received information about AMR by playing a board game aiming to improve AMR knowledge. The participants in the control group (CG) received the same information but by a conventional lecture. This study has been documented using CONSORT guidelines. The participants were female volunteers recruited from the female department of Friends Association Charitable Society (FACS) in Makkah, Saudi Arabia. Inclusion criteria were (a) Arabic speakers, (b) older than 18 years old, and (c) agreement to participate in the intervention and answer all of the questionnaires. Potential participants who did not agree to sign the consent form were excluded from the study. The invitations were sent using the mobile phone database from the female department of FACS listing only active members who had attended most activities for FACS (*n* = 112), with the same message.

The sample size was calculation using RCT with two independent samples, continuous outcomes and two tailed hypothesis formula [15]:

$$ n\left( per\  group\right)=2{\left(\frac{Z_{1-\alpha /2}+{Z}_{\upbeta -1}}{ES}\right)}^2 $$

$$ ES=\left(\frac{\mathrm{minimal}\ \mathrm{clinical}\ \mathrm{difference}}{standard\ devation}\right) $$

The ES means effect size, the value of α = 0.05, β (study power of 90%) = 0.1, constant Z (1- α/2) = 1.96, and constant Z(β-1) = 1.282, standard deviation (SD) of previous study = 2.1 [8], and minimal clinical difference of 2 points, were used to result in minimum number of needed participants in each group 23, and 46 participants for both SG and CG (after roundation). The previous number was multiplied by 1.5 for the design effect due to multiple follow up (50%) and by 1.5 again for estimated non-response rate (50%), yielding in approximately 104 invitations needed for this study.

### Setting

All those who agreed to participate in the study registered at the FACS main office and signed the study consent form. The participants were then randomly assigned by the research team into either the SG or the CG. A simple randomization process was used with previously shuffled sealed envelopes with an equal allocation ratio using pieces of paper in a bowl, so that each participant randomly picked out an envelope, resulting in an equal chance of being assigned into one of the two groups. To fulfill concealment of allocation, the sealed envelopes were opaque and numbered sequentially. To ensure blindness, the participants were informed that the study aimed to compare two methods of information delivery meant to improve AMR knowledge. None of the participants was aware that gamification was the main point of interest in the study. Thus, the study was single-blinded. The participants were evaluated three times: (T1) was immediately before the intervention was conducted, (T2) was immediately after the intervention, and (T3) was one month after the intervention. Questionnaires in a self-reported hard copy format were administered at T1, T2, and T3. Participants who did not attend the FACS at T3 were contacted so that a member of the research team could complete the questionnaire by phone. All identifiable data were destroyed after completing T3 data collection.

### Intervention and control

**Fig. 1:**
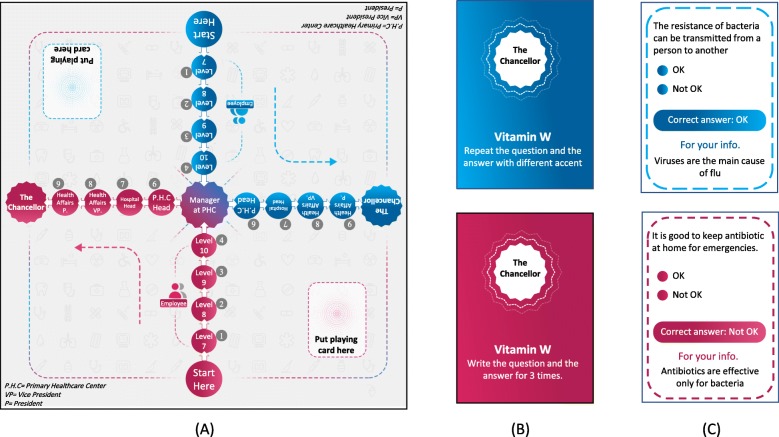
The Chancellor board game layout. (**a**) Board design; (**b**) faces of the cards; and (**c**) backs of the cards. Note: text was translated for publication purposes only

**Fig. 2:**
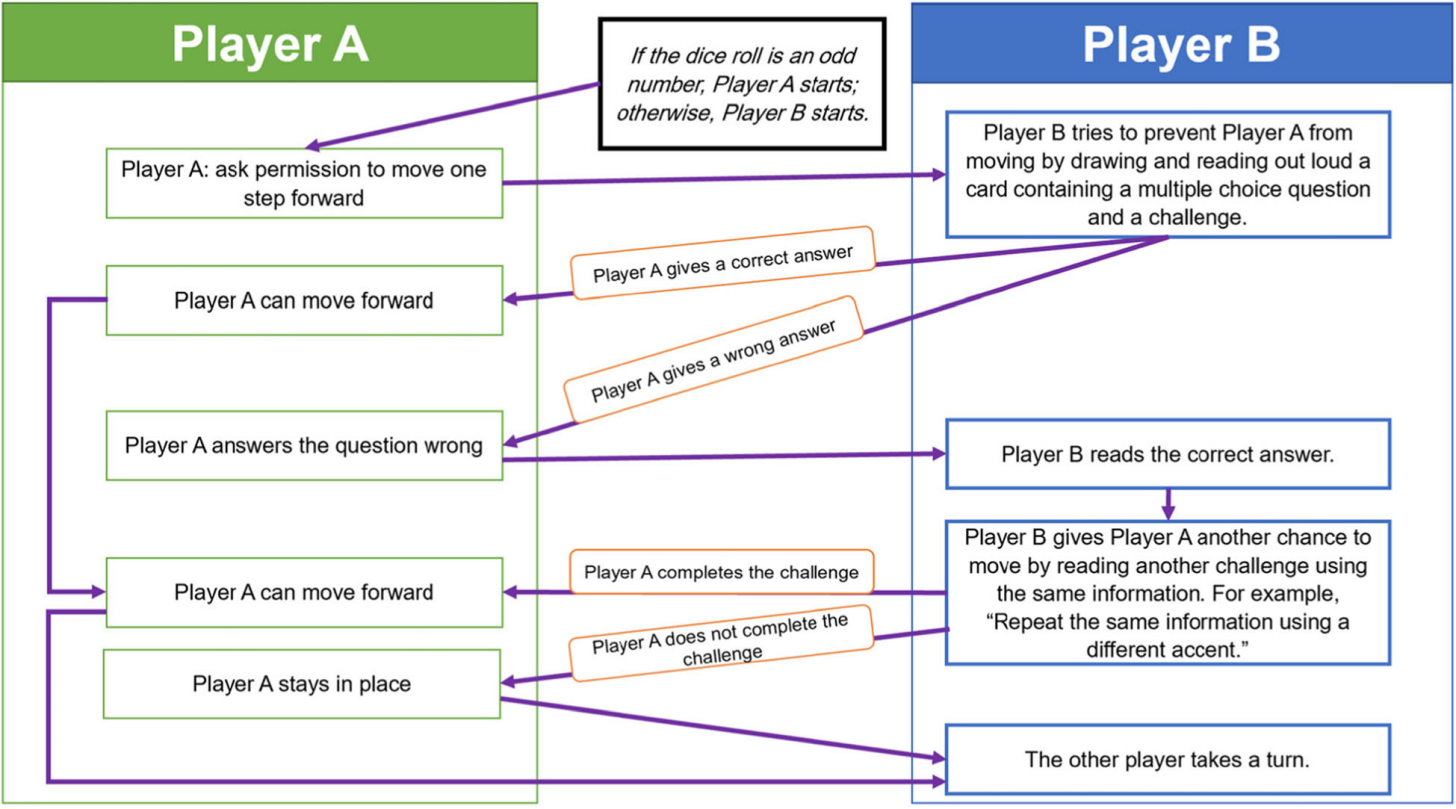
Flow chart of play for The Chancellor game

Participants in the SG played a custom-made educational board game created by the research team, called The Chancellor. The game was created after reviewing most of the popular board games from the website www.boardgamegeek.com with a focus on methods of play (the game mechanism). The game went through three rounds of pilot testing of the gaming experience with groups of 5 to 7 people until reaching the final version to be used in the study. In its final form, the game was played with two players (A and B), with each trying to finish 10 steps on the game board before their opponent. The game is composed of one board, two piles of flash cards, two different pawns for each player, and a pair of dice to decide who plays first (game shown in Fig. 1). At each turn, a player tries to move one step forward, and the opponent tries to stop the opponent’s move by drawing a flash card with a question about AMR, extra information about AMR, and/or a funny challenge. The funny challenge on each card was a request to complete a specific task such as repeating some information about AMR in a different accent or while holding their nose shut. If the player won the challenge, they moved their pawn forward; otherwise, their pawn stayed at the same place, waiting for another turn. The game mechanism is detailed in Fig. 2. Each game took around 20 to 30 min. Participants played the game at the same time in multiple sets of two players. For more details about the game, you can contact the study authors. During the intervention, the game was supervised by the research team, who offered explanations and facilitated play.

At the same time, the CG received a lecture entitled “Antimicrobial Resistance,” which consisted of a PowerPoint presentation given by a member of the research team in Arabic. The 20-min was first conducted in a pilot of 10 participants to validate the content, spelling, organization, grammar, syntax, clarity of the questions and listener understanding.

The interventional game and control lecture were delivered at FACS in their theaters and meeting rooms. Both groups were given identical information about AMR, using content that was retrieved from previous studies [16, 17] and other AMR information related to dental treatments [18]. The information included the proper way to store AB prescriptions at home, problems with AB self-prescribing, the relationship of ABs to bacteria and viruses, proper AB indications, ABs and recovery time, ABs and normal gut flora, AB side effect management, natural antibodies, AMR, ABs and embryonic dental development, ABs and dental management, and ABs and dental extractions [16–18].

### Assessment

Hard copies of self-administrated questionnaires testing participants’ knowledge were given at the three time points previously mentioned, T1, T2, and T3. In addition to questions related to AMR and dentistry, some questions were derived from previous studies [16–18]. The questionnaire was divided into three sections: demographics, AMR knowledge, and game experience. The first section’s demographic questions included age, marital status, educational level, and family income. The second section included questions about AMR in general and, more specifically, AMR in relation to dentistry. These questions were answered from a choice of “Agree,” “Do not agree,” and “I do not know.” Questions in section two were scored as correct or incorrect, and the total score of correct answers was summed into the total knowledge score. The third section was administered only for the SG and assessed the participants’ experiences and perceptions of the game’s usability and engagement. This section contained 10 statements, with answers ranging from 1 to 10, where 1 = strongly disagree and 10 = strongly agree. Some of the questions in section three were derived from a previous article [8], with modifications, while the rest were created by the research team. All three sections of the questionnaire were administrated in Arabic and were face and content validated during a pilot with 10 participants.

### Incentives and ethical considerations

All identifiable data were destroyed after data collection at T3 was completed. The participants each received certificates of appreciation after completing the follow-ups. They were also entered into two random prize drawings for 50 Saudi Riyal (USD 13.33) in the form of vouchers from a local bookstore. Participation was completely voluntary and unpaid, and all participants signed the study consent form before taking part in the intervention. The consent included all information about the RCT including the the three times assessments. Formal approval was received from FACS, and ethical approval was received from the faculty of dentistry at Umm Al-Qura University ethics committee, with number 120–19. The study was registered in ISRCTN with registry number ISRCTN15884410.

### Data analysis

The data were collected, tabulated, and statistically analyzed using SPSS software package version 21 (IBM Corp., Armonk, NY, USA). Chi-square, Fisher’s exact test, t-test, and paired t-test were used in the analysis.

## Results

**Fig. 3:**
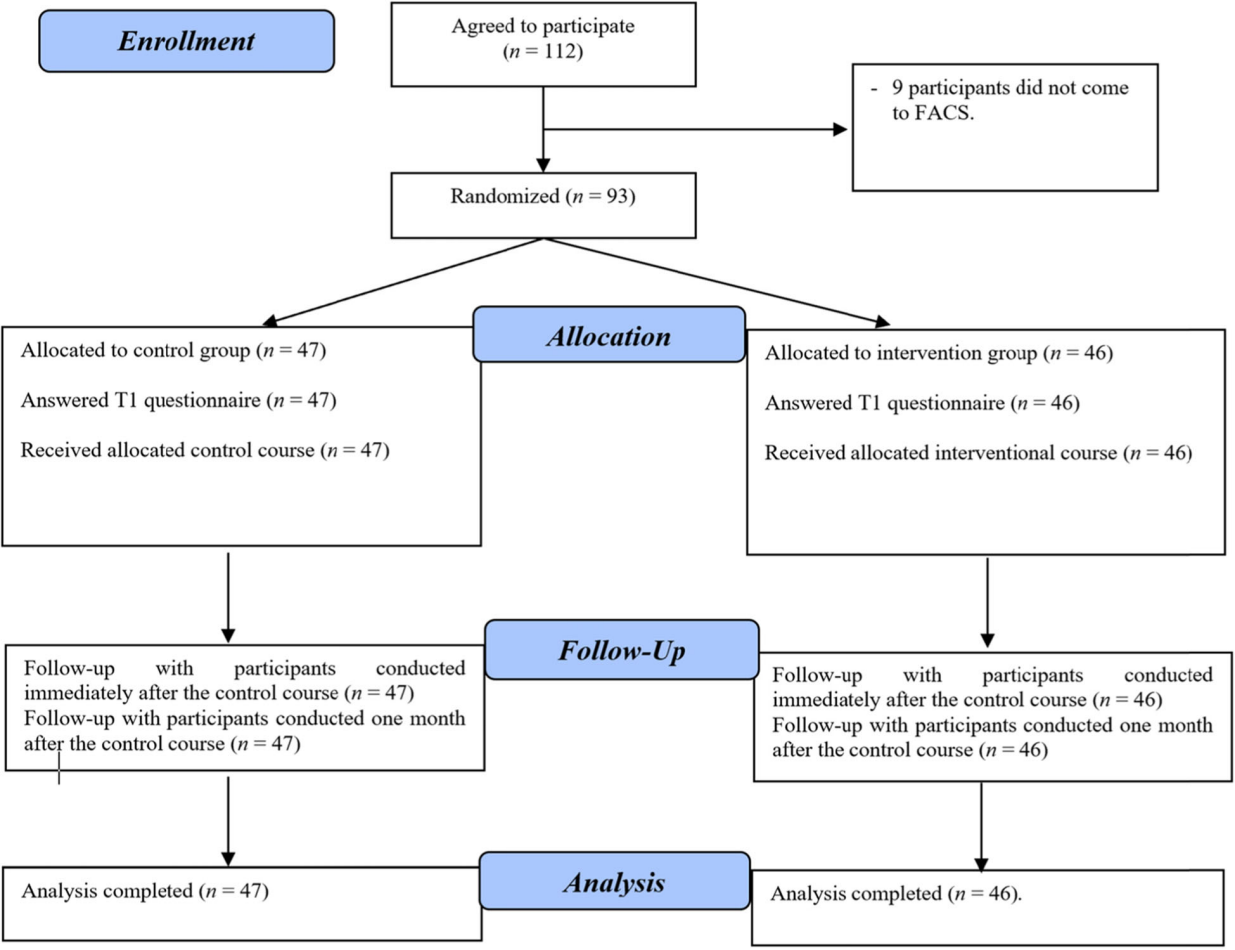
Flow of participants through the randomized controlled trial

Out of the 112 individuals invited to participate in the study, only nine refused to participate (response rate = 83.3%), so a total of 93 people participated in this study. After randomization, there were 46 participants in SG and 47 participants in CG. All of the participants answered the T1, T2, and T3 questionnaires, with no dropouts, as shown in Fig. 3. In addition, there were no missing values. The mean age of the participants was 28.13, with standard deviation (SD) of 9.19 years. Table 1 shows the participants’ demographic data.

**Table 1:** Participant demographic information

Variable		***n***	%
Income (Saudi Riyal)	Less than 5000	61	65.5%
	5000–10,000	19	20.4%
	More than 10,000	13	14.1%
Marital status	Married	17	18.3%
	Unmarried	76	81.7%
Educational level	Middle school or less	10	10.8%
	High school	40	43.0%
	Undergraduate or more	43	46.2%

The chi-square, Fisher’s exact test, and t-test analyses showed no significant differences between the SG and the CG for any demographic variable.

Table 2 shows the mean of the total knowledge score, SD, and minimum and maximum values for the CG and SG at T1, T2, and T3. The minimum possible score was zero, and the highest possible score was 27.

**Table 2:** The knowledge mean of control and study groups at T1, T2, and T3

		Mean	SD	Maximum	Minimum
Study	T1	13.95	4.03	22	5
	T2	21.60	3.15	27	14
	T3	19.78	3.32	24	20
Control	T1	14.25	3.52	21	7
	T2	20.30	3.61	26	10
	T3	16.17	3.40	24	10

A t-test analysis showed no significant differences in the total knowledge scores of the SG and CG at T1 or T2. However, the SG score was significantly higher than the CG at T3 (t (91) = 5.176, *p* <  0.001).

The results of a paired t-test showed that the SG total knowledge scores increased significantly from T1 to T2 (t (45) = − 11.995, *p* < 0.001), decreased significantly from T2 to T3 (t (45) = 3.634, *p* = 0.001), and were significantly higher at T3 than at T1 (t (45) = − 8.346, *p* < 0.001). The results were the same for the CG, where they increased significantly from T1 to T2 (t (46) = − 10.721, *p* < 0.001), decreased significantly from T2 to T3 (t (46) = 8.28, *p* < 0.001), and were significantly higher at T3 than at T1 (t (46) = − 3.055, *p* = 0.004).

**Fig. 4:**
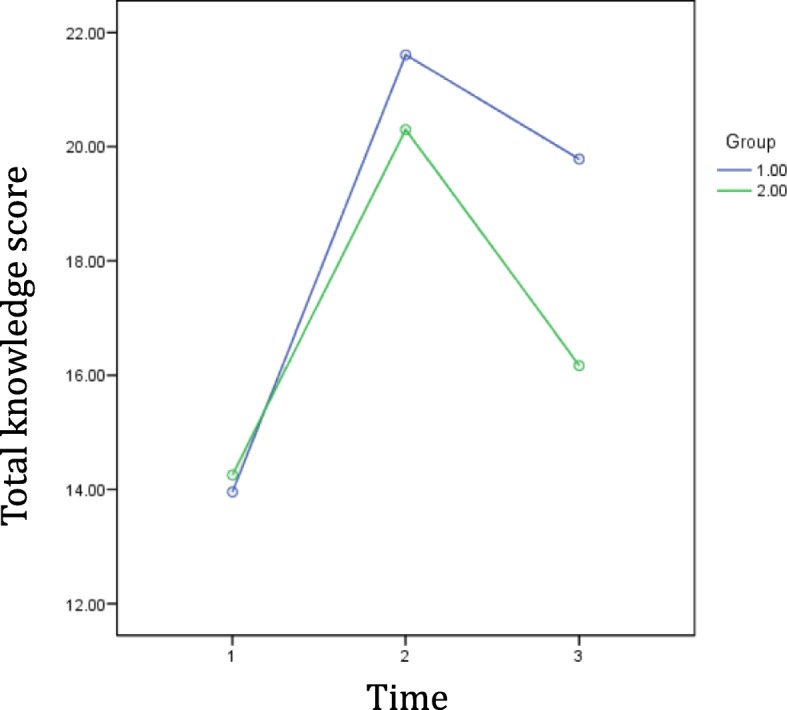
Changes in total knowledge score over time

Table 3 shows the difference in total knowledge scores between T1, T2, and T3. The t-test analyses showed that the improvements in scores from T1 to T2 were not significantly different between the SG and the CG (t (89.408) = − 1.89, *p* = 0.062). However, the reduction of total knowledge scores from T2 to T3 was significantly greater in the CG than in the SG (t (90.967) = − 3.252, *p* = 0.002). Also, the overall improvements in total knowledge scores from T1 to T3 were significantly higher in the SG (t (89.749) = − 4.169, *p* < 0.001). Figure 4 shows the trajectory of the total knowledge scores.

**Table 3:** Differences in knowledge scores at T1, T2, and T3

	Study Group m (SD)	Control Group m (SD)	***p***-value
Difference from T1 to T2	7.65 (4.33)	6.04 (3.87)	0.062
Difference from T2 to T3	−1.83 (3.41)	−4.13 (3.42)	0.002
Difference from T1 to T3	5.83 (4.73)	1.91 (4.30)	< 0.001

Table 4 shows the questions regarding participant experiences with and opinions about the intervention game that were asked only in the questionnaires for participants in the SG (*n* = 46).

**Table 4:** Participant experiences with and opinions about the game as an intervention

	M	SD
I am generally fully satisfied with the game.	9.87	0.54
I found the game enjoyable.	9.85	0.56
The game contains useful information.	9.96	0.21
The time needed to complete the game is appropriate.	9.91	0.35
The game rules were easy to understand.	9.93	0.33
The game colors and design were appropriate.	9.78	0.76
This game is competitive.	9.65	1.42
The game was motivating.	9.70	0.99
I would recommend the game to other people.	9.59	1.53
I could play a game using the same rules but with different content.	9.63	1.31
I found the game to be suitable for my age.	9.67	1.03

## Discussion

This study aimed to assess the effects of gamification using a board game on levels of knowledge about AMR among a sample of female adults in Saudi Arabia. The results indicated that the educational board game contributed to a significant level of knowledge improvement and significant retention of information one month after the intervention as compared to a control group.

In fact, our study results were, in general, similar to previous interventional studies [6, 8, 14], and they support the main results that gamification is an effective method to be used for improving knowledge about not only AMR but also other aspects of knowledge about oral health. This is despite differences in the different study settings and in other aspects, as detailed below.

The previous study by Hale et al. [14] was the only other prior study to use gamification in regard to AMR. The researchers in that study used three different online games for children and made the assessment immediately after participants finished that game. The study results showed that the children enjoyed the games at various levels, according to the game style. Our study was different in that it used a board game, which is more easily used by people with no access to the internet, and is likely to be less expensive and requires no programming. Nevertheless, it might be less accessible for applying to mass educational efforts. Our study assessed the retention of information at a longer follow-up time point. In addition, our study has another advantage in comparing the results with a control group who received the same information in a lecture format. Both studies were found to be enjoyable by the child and adult participants, respectively, which can indicate that games used for health promotional purposes are valid for use with children and adults, as well as for different intellectual levels and interests. We should also state that our study included a focus on AMR in relation to oral health care, which was not included in Hale et al.’s study or any other similar intervention.

The study by El Tantawi et al. reported an improvement in dental student writing skills after the intervention, but there were low satisfaction levels [8]. Satisfaction levels were high in our study, and participants rated the game highly in terms of recommending it to others. Participants also considered the game suitable for their age group. This might be due to differences between our game and the interventional game used by El Tantawi et al. [8], which gave priority to the inclusion of a high volume of information and complex level of academic writing skills over a positive game experience. This is the opposite of our intervention, which resulted in a moderate volume of information after many attempts to make the game a positive experience for participants. This could indicate that the volume of information, complexity of the information, and game experience are important factors for achieving the desired improvement.

A third study, by Saraswathy [6], was similar to ours in using a board game and finding positive outcomes. However, the Saraswathy study was conducted with children and measured the improvement immediately after the completion of the seven days of intervention. Our findings indicated that the board game could be played once and still have a good impact among adults, with longer retention of the information. However, we argue that this can occur if there was a high level of engagement and enjoyment by participants, as might be explained by Csikszentmihalyi’s flow theory [19–21], which states that if a person is engaged in an experience, there will often be higher levels of immersion and concentration on the task.

Based on the results from our study and previous studies in the literature, we claim that, used properly, gamification can be a useful tool for promoting education about AMR and oral health care. Furthermore, our game design is simple, and the information used can be easily replaced by others to promote education about other oral problems. Nevertheless, more studies are needed to validate this argument.

Also, it should be noted that most of the previous studies aiming to improve awareness about AMR were conducted in high-income countries [13]. Our gamification tool using a board game can be an additional method used to improve AMR awareness and can be used in developed countries or undeveloped countries and for people living in socioeconomically poor areas, as in our study, avoiding the barriers to access that comes from a lack of access to the internet or computers.

As most of the previous studies used gamification for specific group of people, this study aimed to assess the effect of gamification on general population to fill the gap in literature. Also, only females were invited to participate. The reason for this is that there are traditional barriers between male and female in Makkah city to do some activities such as playing a board game, despite the social changes accompanying Saudi Arabia Vision 2030 initiatives. So, it was more convenient to involve either male of female in this study. Female were chosen because they usually in charge with children medication in Saudi families. However, this can reduce the external validity of this study results. A further study is needed to include both male and female to re-evaluate gamification and its effectiveness in AMR knowledge improvement.

This study had several strengths, including the single-blinded RCT design and 0% drop-out rate. In fact, this study is considered the first RCT in Saudi Arabia that aimed to assess the effectiveness of gamification for improving knowledge and attitudes about AMR in relation to dentistry. However, a number of limitations should be acknowledged, including all participants being female, a small sample size, and the study being conducted in only one center. In fact, involving participants only from FACS, reduces the external validity for results to be applied on general population easily. Also, longer follow-up time lines are needed to validate the length of retention of the information. Further study is recommended, using larger sample sizes and multiple centers, for more generalizable results. Further study is needed using different sample representing general population in Saudi Arabia to give external validity to the results. It would also be valuable to use different content in the game to assess its effectiveness on other areas of oral health.

## Conclusion

Gamification using board games seems to be a promising tool for promotional efforts to improve public health knowledge about AMR in relation to dental treatment, as well as other oral health care topics. A board game is easy and affordable for use in middle and low socioeconomic communities, and this game provided good levels of retention of information about AMR. Nevertheless, further studies are needed to generalize this study’s results.

## Data Availability

The data set analyzed during this study is available from the corresponding author upon reasonable request.

## Abbreviations

AMR: Antimicrobial resistance
AB: Antibiotics
RCT: Randomized controlled trial
T1: Time of assessment before the intervention
T2: Time of assessment immediately following the intervention
T3: Time of assessment one month after the intervention
WHO: World Health Organization
FACS: Friends Association Charitable Society
SG: Study group
CG: Control group
SD: Standard deviation

## References

[1] Hamari J, Koivisto J, Sarsa H. Does gamification work? – A literature review of empirical studies on gamification. In: 2014 47th Hawaii international conference on system sciences (HICSS). Hawaii: IEEE; 2014. p. 3025–34. 10.1109/HICSS.2014.377.

[2] Chen H, Jian C, Lin W, Yang P, Chang H. Design of digital game-based learning in elementary school mathematics. In: 2014 7th international conference on Ubi-Media computing and workshops (UMEDIA). Ulaanbaatar: IEEE. p. 322–5. 10.1109/U-MEDIA.2014.29.

[3] Barata G, Gama S, Jorge J, Gonçalves D. Engaging engineering students with gamification. In: 2013 5th international conference on games and virtual worlds for serious applications (VS-GAMES). Poole: IEEE; 2013. p. 1–8. 10.1109/VS-GAMES.2013.6624228.

[4] Nevin CR, Westfall AO, Rodriguez JM, Dempsey DM, Cherrington A, Roy B, Patel M, Willig JH (2014). Gamification as a tool for enhancing graduate medical education. Postgrad Med J.

[5] Shiyko M, Hallinan S, Seif El-Nasr M, Subramanian S, Castaneda-Sceppa C (2016). Effects of playing a serious computer game on body mass index and nutrition knowledge in women. JMIR Serious Games.

[6] Saraswathy J (2012). Effectiveness of snake and ladder game on level of knowledge regarding oral hygiene among school children in selected schools, Salem [dissertation].

[7] Kumar Y, Asokan S, John B, Gopalan T (2015). Effect of conventional and game-based teaching on oral health status of children: a randomized controlled trial. Int J Clin Pediatr Dent.

[8] El Tantawi M, Sadaf S, AlHumaid J (2018). Using gamification to develop academic writing skills in dental undergraduate students. Eur J Dent Educ.

[9] World Health Organization (2014). Antimicrobial resistance: global report on surveillance.

[10] Morgan DJ, Okeke IN, Laxminarayan R, Perencevich EN, Weisenberg S (2011). Non-prescription antimicrobial use worldwide: a systematic review. Lancet Infect Dis.

[11] Belkina T, Al Warafi A, Hussein Eltom E, Tadjieva N, Kubena A, Vlcek J (2014). Antibiotic use and knowledge in the community of Yemen, Saudi Arabia, and Uzbekistan. J Infect Dev Ctries.

[12] Aidasani B, Solanki M, Khetarpal S, Ravi Pratap S. Antibiotics: their use and misuse in paediatric dentistry. A systematic review. Eur J Paediatr Dent. 2019;20(2):133–138; doi:10.23804/ejpd.2019.20.02.10. 10.23804/ejpd.2019.20.02.10 31246090

[13] Price L, Gozdzielewska L, Young M, Smith F, MacDonald J, McParland J, Williams L, Langdridge D, Davis M, Flowers P (2018). Effectiveness of interventions to improve the public’s antimicrobial resistance awareness and behaviours associated with prudent use of antimicrobials: a systematic review. J Antimicrob Chemother.

[14] Hale AR, Young VL, Grand A, McNulty CA (2017). Can gaming increase antibiotic awareness in children? A mixed-methods approach. JMIR Serious Games.

[15] Sullivan LM (2011). Essentials of biostatistics in public health.

[16] McNulty CA, Boyle P, Nichols T, Clappison P, Davey P (2007). Don’t wear me out—the public’s knowledge of and attitudes to antibiotic use. J Antimicrob Chemother.

[17] André M, Vernby Å, Berg J, Lundborg CS (2010). A survey of public knowledge and awareness related to antibiotic use and resistance in Sweden. J Antimicrob Chemother.

[18] Palmer N, Martin M (2016). Antimicrobial prescribing for general dental practitioners.

[19] Chen J (2007). Flow in games (and everything else). Comm ACM.

[20] Csikszentmihalyi M (1997). Creativity: flow and the psychology of discovery and invention.

[21] Nakamura J, Csikszentmihalyi M, Snyder C, Lopez SJ (2002). The concept of flow. Handbook of positive psychology.

